# Traction Devices May Not Affect the Vertical Margin Distance in the Endoscopic Submucosal Dissection of Rectal Neuroendocrine Tumors

**DOI:** 10.7759/cureus.58976

**Published:** 2024-04-25

**Authors:** Junnosuke Hayasaka, Yasuro Miura, Satoshi Yamashita, Akira Matsui, Daisuke Kikuchi, Yutaka Takazawa, Shu Hoteya

**Affiliations:** 1 Gastroenterology, Toranomon Hospital, Tokyo, JPN; 2 Pathology, Toranomon Hospital, Tokyo, JPN

**Keywords:** vertical margin, rectum, endoscopic submucosal dissection, traction device, neuroendocrine tumor

## Abstract

Introduction

The usefulness of traction devices (TDs) in endoscopic submucosal dissection (ESD) for rectal neuroendocrine tumors (NETs) has not been reported. The aim of this study was to investigate the impact of using a TD on the vertical margin (VM) distance in the ESD of rectal NETs.

Methods

In this single-center, retrospective study, we included patients with rectal NETs who were treated with ESD during 2013-2023. They were divided into TD and non-TD groups. One pathologist remeasured the VM distance (primary outcome) and the depth of submucosal invasion (SM depth). Secondary outcomes were margins, resection time, delayed bleeding, and perforation. First, we performed propensity score matching (PSM) to assess the usefulness of TD for VM distance. Then, we used multiple regression analysis to identify factors affecting the VM distance.

Results

The TD and non-TD groups comprised 24 and 117 lesions, respectively. Patients in the TD group were significantly younger than those in the non-TD group (P = 0.003). In the TD and non-TD groups, the VM distance was 150 μm and 100 μm, respectively (P = 0.70). Only resection time significantly differed between groups, shorter in the TD group (P = 0.005). Twenty-two cases in each group were matched after PSM, yielding no significant differences in VM distance. The use of a TD was not an independent predictor of VM distance (P = 0.65), but age (P < 0.001) and SM depth (P = 0.003) were.

Conclusion

Using a TD does not seem to affect the VM distance in ESD for rectal NETs.

## Introduction

Rectal neuroendocrine tumors (NETs) are rare but increasing in frequency [1,2]. Furthermore, rectal NETs are increasingly diagnosed at an early stage [2]. This may be due to the widespread use of screening colonoscopy [3]. Therefore, the treatment of rectal NETs in the early stages is expected to increase in the future, along with the demand for endoscopic treatment.

In Japan, endoscopic treatment is indicated for rectal NETs that are less than 10 mm wide, designated a World Health Organization (WHO) classification of no higher than grade 1, and remain in the submucosa without metastasis [4]. Endoscopic treatment for rectal NETs includes conventional endoscopic mucosal resection (EMR), cap-assisted EMR, endoscopic submucosal resection with a ligation device, and endoscopic submucosal dissection (ESD) [5-7]. EMR has the advantage of simplicity and a short resection time but the disadvantage of a low complete resection rate and R0 resection rate compared to ESD [6-10]. As most NETs extend into the submucosal layer and require resection in deeper layers than intramucosal lesions, ESD, with its ability to adjust the dissection layer, is considered more suitable for treatment than conventional EMR. However, the dissected layer cannot always be preserved with ESD, resulting in a positive vertical margin (VM). In such cases, additional surgical resection should be considered; however, rectal surgery has a significant impact on a patient’s quality of life, including causing defecation problems. Therefore, a higher negative VM rate is required with ESD.

Recent reports have indicated that colorectal ESD can be simplified using a traction device (TD) [11-13]. The use of a TD for ESD of rectal NETs may increase the depth of dissection [14,15]. However, this possibility has to be verified. In this study, we aimed to determine the utility of a TD in ESD for rectal NETs in terms of the depth of dissection and to identify factors affecting the depth of dissection.

## Materials and methods

In this retrospective study, we included cases of ESD for rectal NETs conducted from January 2013 to August 2023 and for which pathological re-evaluation was possible. In total, 152 rectal NETs were endoscopically resected at Toranomon Hospital, Tokyo, Japan, during this period, all treated with ESD. Four lesions were excluded because ESD was the additional treatment of a positive VM from other endoscopic treatments or because no tumor was detected upon pathological examination. Two lesions on which the pocket-creation method was performed and five lesions on which the clip-thread method was performed were excluded. The latter were excluded because the clip-thread method was used when conventional ESD was difficult to perform. When multiple lesions were observed in a specimen, the lesion with the shallowest dissection depth was selected. The remaining 141 lesions were divided into TD and non-TD groups and retrospectively compared. Two types of TD were used in the TD group: an S-O clip (Zeon Medical Co. Ltd., Tokyo, Japan) and a multi-loop TD (Boston Scientific Co. Ltd., Tokyo, Japan).

**Figure 1:**
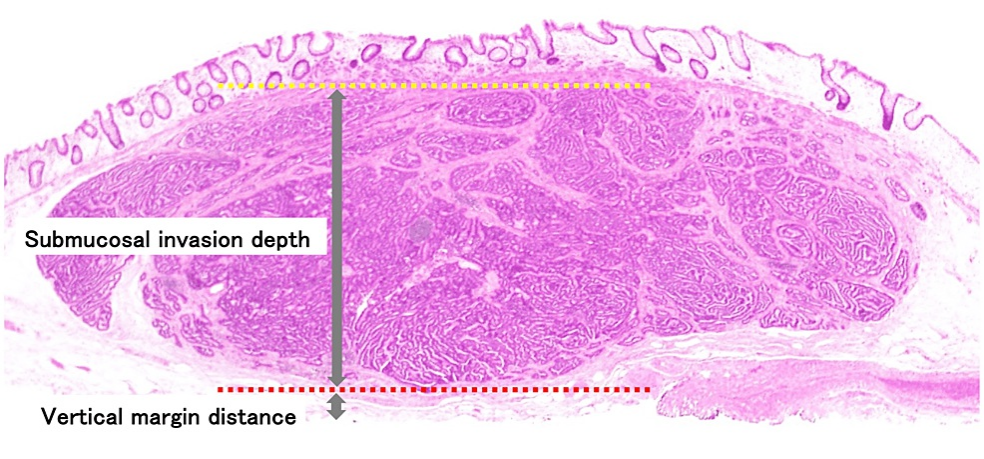
The vertical margin distance and submucosal invasion depth measurements displayed with hematoxylin-eosin staining. The yellow dotted line indicates the lower edge of muscularis mucosa. The red dotted line indicates the invasion deepest point.

We retrospectively collected the following information from the patients’ medical records: age, sex, body mass index (BMI), the physician’s experience (number of colorectal ESDs performed), use of hyaluronic acid, resection time, delayed bleeding, perforation, lesion location, degree of submucosal fibrosis, lesion size, NET grade, lymphovascular invasion, and margins. One pathologist (Y.M.), blinded to clinical information in this study, measured the VM distance in the resected specimen and the invasion depth to the submucosal layer. We defined the invasion depth to the submucosal layer as the submucosal invasion depth (SM depth) measured from the lower edge of the muscularis mucosa to the invasion's deepest point. Figure 1 demonstrates the actual measurement method. A positive VM was defined as exposure of the tumor to the submucosal VM of the resected specimen, with a measurement distance of 0 μm. A physician who had performed less than 50 colorectal ESD procedures was defined as a non-expert in colorectal ESD.

The following is a summary of the conventional ESD and the ESD with a TD performed at our institution. The use of a TD during ESD was performed at the discretion of the physician.

Conventional ESD

Marking dots are placed around the lesion, with margins of approximately 5 mm. After submucosal injection of a glycerol solution containing indigo carmine, a mucosal incision is made on the outside of the marker dots. The incision is initiated at either the oral or the anorectal side of the tumor, dissection is performed in the same direction as the starting incision, and a full circumferential incision is made after more than half of the dissection is completed. Thereafter, the dissection is resumed, and the tumor is resected.

ESD with TD

**Figure 2:**
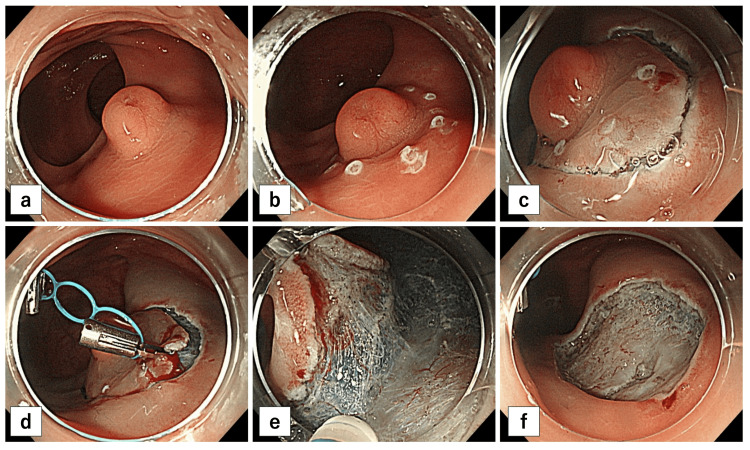
Endoscopic submucosal dissection with a multi-loop traction device. (a) Neuroendocrine tumor in the rectum (9 mm in diameter) in the Rb location. (b) Marker dots placed around the lesion. (c) Full circumferential incision before dissection. (d) Traction using a multi-loop traction device. (e) Dissection with the multi-loop traction device. (f) Ulcer after endoscopic submucosal dissection.

Figure 2 illustrates ESD with a TD. Marking and submucosal injections are performed as for conventional ESD (Figure 2b). Unlike in conventional ESD, a full circumferential incision is made first (Figure 2c). Before dissection, the TD is placed on the anorectal side of the lesion, and traction is applied to the opposite side (Figure 2d). The TD is clipped at a point where moderate traction is applied from the anal side of the lesion to the oral side of the healthy colon tissue to the anorectal from the oral side of the healthy colon tissue. Subsequently, dissection and resection are performed in the same direction as the circumferential incision (Figure 2e), and the tumor is resected (Figure 2f).

The primary outcome was the VM distance. Secondary outcomes were horizontal and vertical margins, resection time, delayed bleeding, and perforation. The resection time was measured from the start of the incision to the end of the dissection. Delayed bleeding was defined as gross hematochezia requiring endoscopic hemostasis or a decrease in hemoglobin of ≥2 g/dl. Perforation was defined as the presence of free air observed upon radiography or computed tomography.

Statistical analysis

Continuous variables are presented as medians and were compared using the Wilcoxon rank-sum test. Categorical variables are presented as percentages and were compared using the chi-squared test or Fisher’s exact test. Propensity score matching (PSM) was performed to minimize the selection bias. Logistic regression analysis was used to calculate propensity scores, and 1:1 matching was performed using nearest-neighbor matching and caliper widths of 0.2 standard deviations. Factors considered clinically important, such as age, sex, BMI, physician experience, use of hyaluronic acid, lesion location, and lesion size, were included in the propensity score calculation. A similar analysis was performed between nonexpert and expert as a subgroup analysis. Multiple regression analysis was used to identify factors affecting VM distance in the analysis of the primary outcome. In the multiple regression analysis, we adjusted for age, sex, BMI, physician experience, use of hyaluronic acid, lesion location, lesion size, and SM depth as explanatory variables.

Statistical significance was set at P < 0.05. All analyses were performed using R software version 4.1.3 (The R Foundation for Statistical Computing, Vienna, Austria).

Ethical approval

This study was conducted in accordance with the 1975 Declaration of Helsinki (6th edition, 2008) and approved by Toranomon Hospital Ethics Committee (approval number: 2471). Written informed consent was not required for this study because of its retrospective nature. However, patients were notified that they could opt out of the study.

## Results

Clinicopathological characteristics are summarized in Table 1. Patients in the TD group were significantly younger than those in the non-TD group (P = 0.003). Hyaluronic acid was used more frequently in the TD group; however, the difference was not statistically significant (P = 0.066). The location of the lesion was Rb in approximately 80% of cases in each group. According to the WHO classification, more than 90% of the cases in both groups were NET G1, whereas no cases of NET G3 were observed. The SM depth was 1200 μm in the TD group and 1100 μm in the non-TD group, a nonsignificant difference (P = 0.41).

**Table 1:** Clinicopathological background of the TD and non-TD groups Values are presented as n (%) or median (interquartile range). * Fisher's exact test was used. Statistical significance was set at P < 0.05. NET, neuroendocrine tumor; SM depth, submucosal invasion depth; TD, traction device; WHO, World Health Organization; Rb, Rectum (below the peritoneal reflection); Ra, rectum (above the peritoneal reflection); Rs, rectosigmoid

	All patients			Propensity score-matched patients		
	TD group	Non-TD group	P value	TD group	Non-TD group	P value
	n=24	n=117		n=22	n=22	
Age, years	48.0 (42.0, 56.0)	58.0 [49.0 69.0]	0.003	50.5 (44.3, 56.0)	50.0 (41.8, 57.5)	0.17
Sex			0.81			1.00
Male	16 (66.7)	72 (61.5)		15 (68.2)	15 (68.2)	
Female	8 (33.3)	45 (38.5)		7 (31.8)	7 (31.8)	
Body mass index, kg/m^2^	22.5 (21.1, 24.3)	23.2 (20.8, 25.6)	0.35	23.2 (21.9, 24.3)	23.2 (20.1, 25.8)	0.24
Hyaluronic acid	10 (41.7)	25 (21.4)	0.066	10 (45.5)	11 (50.0)	1.00
Non-expert	9 (33.3)	57 (48.7)	0.25	8 (36.4)	11 (50.0)	0.54
Location			1.00*			1.00*
Rb	19 (79.2)	89 (76.1)		17 (77.3)	17 (77.3)	
Ra/Rs	5 (20.8)	28 (24.0)		5 (22.7)	5 (22.7)	
Degree of submucosal fibrosis						
No fibrosis	24 (100)	117 (100)	1.00	22 (100)	22 (100)	1.00
Lesion size, mm	5.0 (3.8, 6.1)	5.0 (4.0, 7.0)	0.28	5.0 (4.0, 6.4)	4.8 (4.0, 5.8)	0.56
WHO Classification			0.20*			0.49*
NET G1	22 (91.7)	114 (97.4)		20 (90.9)	22 (100)	
NET G2	2 (8.3)	3 (2.6)		2 (9.1)	0 (0.0)	
NET G3	0 (0.0)	0 (0.0)		0 (0.0)	0 (0.0)	
Lymphatic invasion	5 (20.8)	18 (15.4)	0.55*	5 (22.7)	3 (13.6)	0.70*
Venous invasion	4 (16.7)	32 (27.4)	0.32*	3 (13.6)	4 (18.2)	1.00*
SM depth, μm	1200 (580, 2000)	1100 (800, 2000)	0.41	1200 (580, 2000)	1050 (830, 1800)	0.75

Primary outcomes

The outcomes of this study are summarized in Table 2. The VM distance was 150 μm in the TD group and 100 μm in the non-TD group, a nonsignificant difference (P = 0.70). Via PSM, 22 cases were matched in each of the TD and non-TD groups. No significant difference in VM distance was observed between the two groups after PSM.

**Table 2:** Outcomes of the TD and non-TD groups Values are presented as n (%) or median (interquartile range). All categorical variables were compared using Fisher’s exact test. Statistical significance was set at P < 0.05. TD, traction device

	All patients			Propensity score-matched patients		
	TD group	Non-TD group	P value	TD group	Non-TD group	P value
	n=24	n=117		n=22	n=22	
Primary outcome						
Vertical margin distance, μm	150 (50, 210)	100 (50, 200)	0.70	150 (50, 240)	100 (63, 200)	0.76
Secondary outcomes						
Horizontal margin, negative	24 (100)	115 (98.3)	1.00	22 (100)	0 (0.0)	1.00
Vertical margin, negative	24 (100)	112 (95.7)	0.59	22 (100)	21 (95.5)	1.00
Resection time, min	24.0 (18.0, 30.0)	31.0 (23.0, 45.0)	0.005	24.0 (18.0, 29.8)	36.0 (23.5, 53.4)	0.018
Delayed bleeding	1 (4.2)	1 (0.9)	0.31	1 (4.5)	0 (0.0)	1.00
Perforation	0 (0.0)	1 (0.9)	1.00	0 (0.0)	0 (0.0)	1.00

Secondary outcomes

No significant differences were observed in horizontal and vertical margin negativity; however, in the TD group, the margins were negative in all cases. Resection time was significantly shorter in the TD group (P = 0.005). The rates of delayed bleeding and perforation did not significantly differ between the groups. After PSM, all outcome results were unchanged, and the resection time remained significantly shorter in the TD group (P = 0.018).

Non-expert

The results are shown in Table 3. The VM distance was 150 μm in the TD group and 100 μm in the non-TD group, a nonsignificant difference (P = 0.17). Resection time was significantly shorter in the TD group (P = 0.031). After PSM, most outcomes remained same, although resection time was shorter in the TD group but not significantly different (P = 0.13).

**Table 3:** Outcomes of the traction and non-traction groups in non-expert Note: Values are presented as n (%), median (interquartile range). All categorical variables were compared using Fisher's exact test. Statistical significance was set at p < 0.05. VMD, vertical margin distance

	All patients			Propensity score-matched patients		
	Traction group	Non-traction group	P value	Traction group	Non-traction group	P value
	N=8	N=57		N=7	N=7	
Primary outcome						
VMD, μm	150 (50, 263)	100 (50, 200)	0.17	100 (50, 250)	200 (100, 225)	0.51
Secondary outcomes						
Horizontal margin, negative	8(100)	115 (98.3)	1.00	7 (100)	7 (100.0)	1.00
Vertical margin, negative	8 (100)	112 (95.7)	1.00	7 (100)	7 (100.0)	1.00
Resection time, min	23.0 (17.5, 27.8)	35.0 (24.0, 52.0)	0.031	26.0 (19.0, 28.5)	42.0 (25.5, 50.0)	0.13
Delayed bleeding	0 (0.0)	1 (1.8)	1.00	1 (4.5)	0 (0.0)	1.00
Perforation	0 (0.0)	0 (0.0)	1.00	0 (0.0)	0 (0.0)	1.00

Expert

The results are shown in Table 4. The VM distance was 150 μm in the TD group and 100 μm in the non-TD group, a nonsignificant difference (P = 0.94). Resection time was shorter in the TD group but not significantly different (P = 0.10). After PSM, most outcomes remained same, although resection time was significantly shorter in the TD group (P = 0.002).

**Table 4:** Outcomes of the traction and non-traction groups in expert Note: Values are presented as n (%), median (interquartile range). All categorical variables were compared using Fisher's exact test. Statistical significance was set at p < 0.05. VMD, vertical margin distance

	All patients			Propensity score-matched patients		
	Traction group	Non traction group	p value	Traction group	Non traction group	p value
	N=16	N=60		N=12	N=12	
Primary outcome						
VMD, μm	150 (50, 200)	100 (50, 200)	0.94	200 (88, 225)	100 (87.5, 200)	0.44
Secondary outcomes						
Horizontal margin, negative	16 (100)	59 (98.3)	1.00	12 (100)	12 (100.0)	1.00
Vertical margin, negative	16 (100)	57 (95.0)	1.00	12 (100)	12 (100.0)	1.00
Resection time, min	25.0 (18.0, 30.0)	29.0 (20.8, 40.0)	0.10	21.0 (17.0, 29.3)	43.5 (31.5, 52.8)	0.002
Delayed bleeding	1 (6.2)	0 (0.0)	0.21	1 (8.3)	0 (0.0)	1.00
Perforation	0 (0.0)	1 (1.7)	1.00	0 (0.0)	0 (0.0)	1.00

Factors affecting VM distance

Multiple linear regression analysis was performed regarding the VM distance (Table 5). TD use was not an independent predictor of VM distance (β = −11.8, P = 0.65), whereas age and SM depth were (β = −2.6, P < 0.001 and β = −0.04, P = 0.003). As the patient’s age and the SM depth increased, the VM distance decreased.

**Table 5:** Multiple linear regression analysis of the vertical margin distance (n = 141) Note: Statistical significance was set at P < 0.05. SM depth, submucosal invasion depth; Rb, rectum (below the peritoneal reflection); Ra, rectum (above the peritoneal reflection); Rs, rectosigmoid

	β	95% confidence interval	P value
Traction device	-11.8	(-62.0, 38.5)	0.65
Age, years	-2.6	(-4.0, -1.1)	<0.001
Sex, male	-19.0	(-56.8, 18.7)	0.32
Body mass index, kg/m^2^	2.0	(-3.1, 0.01)	0.43
Hyaluronic acid	-30.5	(-72.5, 11.4)	0.156
Non-expert	-13.1	(-49.3, 23.2)	0.48
Location, Rb vs Ra/Rs	30.1	(-12.4, 72.7)	0.167
Lesion size, mm	-4.0	(-14.5, 0.01)	0.45
SM depth, μm	-0.04	(-0.06, -0.01)	0.003

## Discussion

In this study, we retrospectively evaluated the effect of using a TD on the dissection depth during ESD for rectal NETs. PSM and multiple regression analyses both revealed that the use of TD did not affect the VM distance. Multiple regression analysis identified only age and SM depth as independent factors affecting the VM distance.

The possibility of TDs being useful in the ESD of NETs was raised in recent reports [14,15]. Using a TD, the field of view of the submucosa can be enlarged and stabilized for dissection. Therefore, we expected the VM distance to be longer to facilitate dissection just above the muscularis, but it did not. However, this study did not support our hypothesis. This may be because the sample of the TD group was small, causing a beta error. Therefore, more cases should be accumulated in the future. A second possible reason is that, as the SM depth increases, the remaining submucosa becomes thinner and the VM distance does not increase, even if its dissection is performed just above the muscle layer. This scenario seems consistent with the results, as multiple regression analysis identified the SM depth as a factor that was negatively associated with the VM distance. Thus, the SM depth was more important than the lesion size because it affected the VM distance. The mean VM distance in the ESD of rectal NETs was previously reported as 202 μm [16]. Although the data in that study were derived from a single institution, our results did not differ significantly from theirs.

Older age was identified as a factor that was negatively associated with the VM distance. Age-related changes and comorbidities, such as arteriosclerosis and submucosal fibrosis, were suspected as causes. However, the ESD results revealed no submucosal fibrosis, regardless of patient age. However, we could not determine whether arteriosclerosis affected ESD in this study. Furthermore, we could find no published evidence on the impact of aging on endoscopic treatment. Therefore, further studies are required to determine whether such an effect exists.

In two randomized clinical trials (RCTs), the use of TDs reduced the procedure time of colorectal ESD [17,18]. Both of those were single-center RCTs, whereas the CONNECT-C trial was the first multicenter RCT in which conventional ESD was compared with ESD with a TD [19]. The results of the latter trial revealed no reduction in procedure time but suggested that it is useful in reducing procedure time when non-experts perform the procedure and when lesions are larger than 30 mm. As that RCT included lesions larger than 20 mm, in contrast to the rectal NETs that were the subject of this study, those results may not be applicable to rectal NETs. In this study, the procedure time was reduced in the univariate analysis. In addition, a reduction in resection time after PSM was observed in the expert group. While a reduction in resection time after PSM was observed in the non-expert group, it was not significant. This difference may be due to the small sample size of the non-expert group. Therefore, ESD with TD for NETs may be useful for both non-expert and expert. This may be attributed to the TD facilitating recognition of the layer to be dissected. Although we are not aware of reports on the use of TDs to increase the rate of negative margins, all patients in the TD group tested negative for resection margins in this study.

The results of this study do not negate the usefulness of TDs, which should be further examined in the future. We plan to investigate whether the use of TDs contributes to shorter procedure times for ESD of rectal NETs.

This study had several limitations. First, it was a single-center, retrospective study, and selection bias was unavoidable. Second, although PSM was used to reduce the effects of selection bias, unmeasured confounders could not be balanced between the two groups. Third, the number of non-experts for whom TD is useful is small and not fully evaluated. Fourth, the pathological diagnosis was made by a single pathologist, so bias is inevitable. Finally, the TD group was small. Despite these limitations, to the best of our knowledge, this is the first study in which the impact of TDs on the VM distance has been examined.

## Conclusions

In this study, the use of a TD did not affect the VM distance in ESD for rectal NETs. Rather, age and SM depth were negatively related to the VM distance. The use of a TD has reduced the resection time for ESD for rectal NETs, and further studies are needed on their usefulness.

## References

[1] Yao JC, Hassan M, Phan A (2008). One hundred years after "carcinoid": epidemiology of and prognostic factors for neuroendocrine tumors in 35,825 cases in the United States. J Clin Oncol.

[2] Dasari A, Shen C, Halperin D (2017). Trends in the Incidence, Prevalence, and Survival Outcomes in Patients With Neuroendocrine Tumors in the United States. JAMA Oncol.

[3] Taghavi S, Jayarajan SN, Powers BD, Davey A, Willis AI (2013). Examining rectal carcinoids in the era of screening colonoscopy: a surveillance, epidemiology, and end results analysis. Dis Colon Rectum.

[4] Ito T, Masui T, Komoto I (2021). JNETS clinical practice guidelines for gastroenteropancreatic neuroendocrine neoplasms: diagnosis, treatment, and follow-up: a synopsis. J Gastroenterol.

[5] Bertani E, Ravizza D, Milione M (2018). Neuroendocrine neoplasms of rectum: A management update. Cancer Treat Rev.

[6] Zhou X, Xie H, Xie L, Li J, Cao W, Fu W (2014). Endoscopic resection therapies for rectal neuroendocrine tumors: a systematic review and meta-analysis. J Gastroenterol Hepatol.

[7] Yong JN, Lim XC, Nistala KR (2021). Endoscopic submucosal dissection versus endoscopic mucosal resection for rectal carcinoid tumor. A meta-analysis and meta-regression with single-arm analysis. J Dig Dis.

[8] Lee DS, Jeon SW, Park SY (2010). The feasibility of endoscopic submucosal dissection for rectal carcinoid tumors: comparison with endoscopic mucosal resection. Endoscopy.

[9] Park HW, Byeon JS, Park YS (2010). Endoscopic submucosal dissection for treatment of rectal carcinoid tumors. Gastrointest Endosc.

[10] Zhong DD, Shao LM, Cai JT (2013). Endoscopic mucosal resection vs endoscopic submucosal dissection for rectal carcinoid tumours: a systematic review and meta-analysis. Colorectal Dis.

[11] Mitsuyoshi Y, Ide D, Ohya TR (2022). Training program using a traction device improves trainees' learning curve of colorectal endoscopic submucosal dissection. Surg Endosc.

[12] Ide D, Saito S, Ohya TR (2019). Colorectal endoscopic submucosal dissection can be efficiently performed by a trainee with use of a simple traction device and expert supervision. Endosc Int Open.

[13] Abe S, Wu SY, Ego M (2020). Efficacy of Current Traction Techniques for Endoscopic Submucosal Dissection. Gut Liver.

[14] Wallenhorst T, Masgnaux LJ, Grimaldi J, Legros R, Rivory J, Jacques J, Pioche M (2023). Obtaining a free vertical margin is challenging in endoscopic submucosal dissection of a rectal neuroendocrine tumor: use of adaptive traction to improve exposure in a child. Endoscopy.

[15] Liu J, Fang N (2023). Traction by dental floss loop for adequate submucosal dissection depth in a rectal neuroendocrine tumor. Endoscopy.

[16] Lim HK, Lee SJ, Baek DH (2019). Resectability of Rectal Neuroendocrine Tumors Using Endoscopic Mucosal Resection with a Ligation Band Device and Endoscopic Submucosal Dissection. Gastroenterol Res Pract.

[17] Ritsuno H, Sakamoto N, Osada T (2014). Prospective clinical trial of traction device-assisted endoscopic submucosal dissection of large superficial colorectal tumors using the S-O clip. Surg Endosc.

[18] Yamasaki Y, Takeuchi Y, Uedo N (2018). Efficacy of traction-assisted colorectal endoscopic submucosal dissection using a clip-and-thread technique: A prospective randomized study. Dig Endosc.

[19] Ichijima R, Ikehara H, Sumida Y (2023). Randomized controlled trial comparing conventional and traction endoscopic submucosal dissection for early colon tumor (CONNECT-C trial). Dig Endosc.

